# Outcomes of uterine sarcoma found incidentally after uterus-preserving surgery for presumed benign disease

**DOI:** 10.1186/s12885-016-2727-x

**Published:** 2016-08-23

**Authors:** Jung-Yun Lee, Hyun Soo Kim, Eun Ji Nam, Sang Wun Kim, Sunghoon Kim, Young Tae Kim

**Affiliations:** 1 Department of Obstetrics and Gynecology, Institute of Women’s Life Medical Science, Yonsei University College of Medicine, 50-1 Yonsei-ro, Seodaemun-gu, 03722 Seoul Korea; 2 Department of Pathology, Yonsei University College of Medicine, Seoul, Korea

**Keywords:** Leiomyosarcoma, Endometrial stromal sarcoma, Myomectomy, Survival analysis, Morcellation

## Abstract

**Background:**

The aims of this study were to evaluate the impact of initial uterus-preserving surgery, such as myomectomy or subtotal hysterectomy, on the recurrence rates of patients with uterine sarcoma found incidentally and to investigate the role of surgical re-exploration in this disease subset.

**Methods:**

We performed a retrospective chart review for patients who had previously undergone either total hysterectomy or subtotal hysterectomy or myomectomy at the time of initial surgery for presumed benign uterine leiomyoma and were found to have uterine sarcoma on final pathology. Survival analysis was performed comparing patients according to the type of initial surgery.

**Results:**

Between 2006 and 2014, 45 patients with uterine sarcoma were identified. Myomectomy or subtotal hysterectomy was performed in 15 patients, and 30 patients underwent total hysterectomy as the initial surgery. Of the patients who underwent myomectomy or subtotal hysterectomy as the initial surgery (*n* = 15), 14 were re-explored to complete staging. Of the patients who underwent re-exploration (*n* = 14), five (35.8 %) had remnant sarcoma on the remaining uterus and no patients had disseminated disease. A Kaplan–Meier curve and log-rank test showed no difference in progression-free survival (*P* = 0.941) between the two groups.

**Conclusion:**

Initial uterus-preserving surgery does not appear to be associated with an adverse impact on survival outcomes for unexpected uterine sarcoma when surgical re-exploration was performed immediately. As such, surgical re-exploration may be useful for removing any remnant sarcoma.

**Electronic supplementary material:**

The online version of this article (doi:10.1186/s12885-016-2727-x) contains supplementary material, which is available to authorized users.

## Background

Uterine leiomyomas are the most common benign uterine tumors [1]. A range of symptoms, from abnormal bleeding to pelvic pressure, are associated with uterine leiomyomas. Surgical management, either myomectomy or hysterectomy, is often required for the management of this form of disease. Surgical options depend on various factors, including age, childbearing requirements and patients’ preferences. Myomectomy is often a good surgical choice for patients of reproductive age who wish to bear children. Furthermore, approximately half of women with leiomyoma prefer uterus-preserving treatment, even after childbearing is completed [2].

Myomectomy or subtotal hysterectomy is the one of the most commonly performed gynecologic surgeries. With recent advances in minimally invasive surgery, laparoscopic myomectomies or hysterectomies have become common practices. Although a major concern with laparoscopic surgery is the removal of large myoma through small incisions, the introduction of morcellation has solved this problem. However, the US Food and Drug Administration (FDA) has issued a statement discouraging the use of power morcellation for hysterectomy and myomectomy due to the fear of potentially disseminating an occult uterine sarcoma [3].

In uterus-preserving surgery, such as myomectomy or subtotal hysterectomy, there may be concerns about tumor aggression, even when morcellation is not used. However, there is limited literature on the management of uterine sarcoma found incidentally after myomectomy or subtotal hysterectomy for presumed uterine leiomyoma [4, 5]. The aims of this study were to evaluate the outcomes of patients with uterine sarcoma found incidentally after initial uterus-preserving surgery for presumed benign disease and to investigate the role of surgical re-exploration in this disease subset.

## Methods

After gaining approval from the Institutional Review Board of Yonsei University Hospital (Registration number: 4-2015-0896), we performed a retrospective chart review for patients who had previously undergone either total hysterectomy or subtotal hysterectomy or myomectomy at the time of initial surgery for presumed benign uterine leiomyoma and were found to have uterine sarcoma on final pathology at the Department of Obstetrics and Gynecology of Yonsei University Hospital, Seoul, Korea between 2006 and 2014.

Study data were collected from patients’ medical charts: age at diagnosis, gravidity, parity, menopausal status, final pathology, stage, type of primary and secondary surgeries, date of surgery, postoperative adjuvant therapy, disease status, location of recurrence and follow-up interval. Intraoperative morcellation was introduced in our department in 2006 as a technique for extracting myomas or the uterus from the abdominal cavity during surgical management. Morcellation techniques include the use of scalpel or scissors in vaginal surgeries (hand morcellation) and a power morcellator in laparoscopy. The open morcellation techinique was performed during the study period, although the in-bag morcellation technique was adopted in 2014. Our institution has since adopted a policy of surgical re-exploration in patients with morcellated sarcoma and/or incomplete surgery, such as myomectomy or subtotal hysterectomy. The type of surgery used at re-exploration depends on each surgeon’s preferences. As a minimum, this includes the removal of the remaining uterus and exploration of the abdominal cavity. Ovarian preservation is considered in young premenopausal women with early-stage sarcomas. Stages are assigned in accordance with the 2009 FIGO staging systems.

All available hematoxylin and eosin-stained slides were reviewed by an independent gynecologic pathologist (H. S. Kim), who was blind to patient outcomes. All endometrial stromal sarcoma cases were diagnosed as low-grade endometrial stromal sarcoma based on characteristic histopathological features and uniform immunoreactivity for CD10, which is an endometrial stromal cell marker.

### Statistical analysis

Descriptive statistics were tabulated by patient group. Continuous variables were summarized by using either standard deviations or medians with ranges. Categorical variables were compared using the chi-square test or Fisher’s exact test. Progression-free survival (PFS) was defined as the time from the date of first treatment to the first occurrence of a local or distant recurrence. PFS was estimated using the Kaplan–Meier method, and differences in survival were compared using the log-rank test. Survival analysis was performed comparing patients according to the type of initial surgery and the use of morcellation.

All analyses were performed using STATA version 12.0 (StataCorp, College Station, TX, US). A two-sided P value of less than 0.05 was considered statistically significant.

## Results

### Patient characteristics

**Fig. 1:**
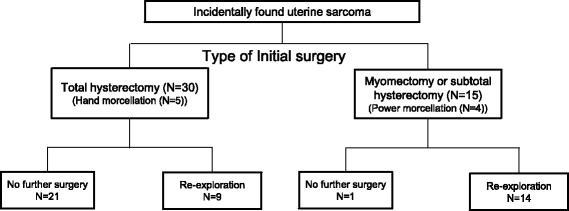
Flow diagram in patients with incidentally found uterine sarcoma

During the study period, 62 patients were diagnosed with uterine sarcoma. Seven of those patients had definite metastatic lesion at diagnosis and 10 underwent staging operations during initial surgery based on the frozen results. As such, 45 patients were diagnosed with unexpected uterine sarcoma after the initial surgery for presumed leiomyoma (Fig. 1). For 22 patients, primary surgery was performed by a general gynecologist at a different institution and the case was then referred to our center after initial management. For 23 patients, surgery was performed by two general gynecologists and five gynecologic oncologists within the Yonsei University Health System. Over two thirds of patients (*n* = 31; 68.9 %) underwent open surgery and 14 underwent laparoscopy (31.1 %). Myomectomy or subtotal hysterectomy were performed in 15 patients (33.3 %), while 30 patients (66.7 %) underwent total hysterectomy as the initial surgery. Of the patients who underwent myomectomy or subtotal hysterectomy as the initial surgery (*n* = 15), 14 (93.3 %) were re-explored to staging operation with total hysterectomy. Of the patients who underwent total hysterectomy as the initial surgery, nine (30.0 %) were re-explored to complete the staging operation. The rate of adjuvant therapy was 33.3 % (5/15) in patients who underwent myomectomy or subtotal hysterectomy and 50 % (15/30) in patients who underwent total hysterectomy.

The baseline characteristics are shown in Table 1. Hand morcellation was performed in five patients who underwent laparoscopic hysterectomy as the initial surgery, while power morcellation was performed in four patients who underwent laparoscopic myomectomy or subtotal hysterectomy as the initial surgery. Of the nine patients with morcellated uterine sarcoma at the initial surgery, one, who had previously undergone hysterectomy, had disseminated disease that was detected during immediate surgical re-exploration. Of the four patients who underwent power morcellation of the uterine sarcoma during the initial uterus-preserving surgery, none were identified as having disseminated disease during immediate surgical re-exploration.

**Table 1:** Patient characteristics

Characteristics	Myomectomy or subtotal hysterectomy (*n* = 15)	Hysterectomy (*n* = 30)
Age (years) median (range)	42 (27–54)	47 (26–66)
Gravidity, median (range)	3 (0–6)	3 (0–6)
≤ 2	6 (40 %)	9 (30 %)
> 2	9 (60 %)	21 (70 %)
Parity, median (range)	2 (0–3)	2 (0–4)
≤ 1	7 (46.7 %)	7 (30.3 %)
> 1	8 (53.3 %)	23 (76.7 %)
Menopause		
Yes	4 (26.7 %)	14 (46.7 %)
No	11 (73.3 %)	16 (53.3 %)
Previous cesarean section		
Yes	3 (20 %)	4 (13.3 %)
No	12 (80 %)	26 (86.7 %)
Mode of initial surgery		
Laparotomy	9 (60 %)	22 (73.3 %)
Laparoscopy	6 (40 %)	8 (26.7 %)
FIGO stage		
IA	5 (33.3 %)	7 (23.3 %)
IB	9 (60.0 %)	22 (73.3 %)
IIA	1 (6.7 %)	1 (3.3 %)
Histology		
Leiomyosarcoma	7 (46.7 %)	11 (36.7 %)
Endometrial stromal sarcoma	7 (46.7 %)	19 (63.3 %)
Adenosarcoma	1 (6.7 %)	0
Morcellation*		
No	11 (73.3 %)	25 (83.3 %)
Hand morcellation	0 (0 %)	5 (16.7 %)
Power morcellation	4 (26.7 %)	0 (0 %)
Surgical re-exploration*		
Yes	14 (93.3 %)	9 (30 %)
No	1 (6.7 %)	21 (70 %)
Adjuvant therapy		
No	10 (66.7 %)	15 (50 %)
Radiation	1 (6.7 %)	5 (16.7 %)^a^
Chemotherapy	4 (26.7 %)	14 (46.7 %)^a^

*P < 0.05

^a^4 patients underwent concurrent chemoradiation

### Survival

The median follow-up duration was 41 months. Among the patients with sarcoma, 10 (18.2 %) experienced recurrence, with a mean time to progression of 11.8 months. Recurrences were localized to the pelvis in three patients and to the paraaortic nodes in one patient, while six patients presented with distant disease (two lung; one hepatic), including two patients with multisite dissemination. During follow-up, five of the patients with sarcoma (50 %) died of the disease.

**Fig. 2:**
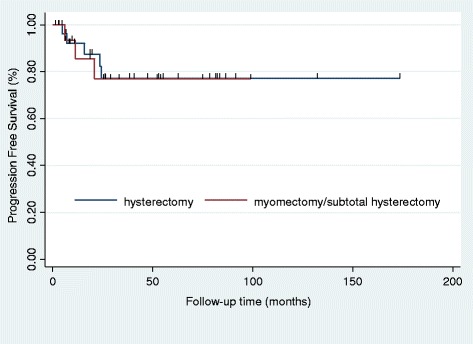
Progression-free survival of patients with unexpected sarcoma stratified by type of initial surgery (myomectomy/subtotal hysterectomy vs. total hysterectomy)

PFS was analyzed according to the type of initial surgery (myomectomy/subtotal hysterectomy vs. hysterectomy) and considering the possibility of tumor aggression in cases of uterus-preserving surgery, even without morcellation. The Kaplan–Meier curves and log-rank test showed no difference in PFS (*P* = 0.941) between patients who underwent myomectomy or subtotal hysterectomy and those who underwent total hysterectomy as the initial surgery (Fig. 2a). We analyzed outcomes in the morcellation and non-morcellation groups and found significant differences in outcomes (*P* = 0.048) (Additional file 1: Figure S1). In the non-morcellation group, recurrence was observed in five patients (13.4 %), while in the morcellation group, recurrence was found in three patients (33.3 %). In the myomectomy/subtotal hysterectomy group, the morcellation subgroup had poorer outcomes than the non-morcellation subgroup (*P* = 0.051) (Additional file 1: Figure S2). In addition, we did subgroup analysis to identify whether survival differences exist between myomectomy and subtotal hysterectomy. We found no significant differences in outcomes (*P* = 0.882) (Additional file 1: Figure S3).

### Role of re-exploration

In the myomectomy/subtotal hysterectomy group, the majority of patients underwent re-exploration to complete the staging operation and remove the remaining uterus. The mean time interval between initial surgery and re-exploration was 18 days. All cases of re-exploration were achieved within 21 days. No major complications were found perioperatively. Five patients (35.8 %) had remnant sarcoma in the remaining uterus and none were upstaged as a result of the staging operation. Ascites or gross metastatic lesions were not found during re-exploration. Detailed information on the patients in these groups is shown in Table 2. Of all the re-exploration cases, 11 (76.8 %) were alive without disease.

**Table 2:** Clinicopathologic features of patients with unexpected uterine sarcoma after myomectomy or subtotal hysterectomy for presumed uterine leiomyoma

Patient	Initial surgery	Site of operation	Morcellation	Histology	Surgical re-exploration	Remnant tumor	State
1	Lap M	participating institution	Yes	LMS	TAH + BSO + Om + PLND + PALND	No	AWD
2	Open M	outside institution	No	LMS	TAH + BSO + Om + PLND + PALND	No	NED
3	Lap subH	outside institution	Yes	LMS	TAH + BSO + PLND	No	D
4	Open subH	outside institution	No	LMS	TAH + BSO + Om + PLND + Appe	No	NED
5	Open M	outside institution	No	LMS	TAH + BSO + Om	No	NED
6	Open subH	outside institution	No	LMS	TAH + BSO + Om + PLND + PALND + Appe	No	NED
7	Open M	outside institution	No	LMS	TAH + LSO + Om + PLND + Appe	No	AWD
8	Open M	outside institution	No	ESS	TAH + BSO + Om + PLND + Appe	Yes	NED
9	Open subH	outside institution	No	ESS	TAH + LSO + Om + PLND + Appe	Yes	NED
10	Lap M	outside institution	Yes	ESS	TLH + LSO + PLND	Yes	NED
11	Lap M	outside institution	Yes	ESS	TLH + BSO + PLND + PALND + Appe	No	NED
12	Open subH	outside institution	No	ESS	TAH + BSO+ Om + PLND + PALND	No	NED
13	Lap M	participating institution	No	AS	TLH + RSO + PLND	Yes	NED
14	Lap M	participating institution	No	ESS	No	N/A	NED
15	Open M	outside institution	No	ESS	TAH + BSO	Yes	NED

*Lap M* laparoscopic myomectomy, *Open M* open myomectomy, *Lap subH* laparoscopic subtotal hysterectomy, *Open subH* Open subtotal hysterectomy, *AWD* alive with disease, *D* death from disease, *NED* no evidence of disease, *TAH* total abdominal hysterectomy, *TLH* total laparoscopic hysterectomy, *BSO* bilateral salpingo-oophorectomy, *LSO* left salpingo-oophorectomy, *RSO* right salpingo-oophorectomy, *Om* omentectomy, *PLND* pelvic lymphadenectomy, *PALND* paraaortic lymphadenectomy, *Appe* appendectomy, *LMS* leiomyosarcoma, *ESS* endometrial stromal sarcoma, *AS* adenosarcoma

## Discussion

In this study, we compared the outcomes of uterine sarcoma found incidentally in terms of the type of initial surgery used (uterus-preserving surgery vs. hysterectomy). Despite concerns about tumor aggression in cases of uterus-preserving surgery, even without morcellation, initial uterus-preserving surgery does not appear to be associated with an adverse impact on survival outcomes for unexpected uterine sarcoma. Immediate surgical re-exploration after uterus-preserving surgery makes it possible to remove remnant sarcoma in the remaining uterus.

Myomectomy is the treatment of choice for uterine myoma when the patient wants to bear children in the future. For women who have completed childbearing, hysterectomy is typically considered to be the surgical treatment of choice for leiomyoma given the risk of recurrence after myomectomy with uterine preservation. However, many women wish to preserve the uterus even after the completion of childbearing. A US survey showed that approximately half of women aged 40–59 believed that uterine preservation was important when considering treatment options for myoma [2]. Many women express concern about the consequences of hysterectomy, including changes to function, emotions and behavior. For these women, myomectomy could be an alternative to hysterectomy, even considering the risk of recurrence or re-operation [6]. Traditional subtotal hysterectomy continues to be performed for a variety of indications, including patient preference and, in patients with challenging anatomy, surgeon preference, reflecting the technical difficulty of removing the cervix [7].

Myomectomy can be performed hysteroscopically, abdominally through a laparotomy, or, more recently, via a minimally invasive surgical approach with laparoscopic or robotic assistance. The removal of large leiomyoma through the small incisions used for minimally invasive myomectomy often poses a challenge. Large leiomyoma can be removed through a small abdominal incision, vaginally by colpotomy or through the use of power morcellation to fragment the leiomyoma. A recent Cochrane review showed that women who underwent minimally invasive surgery had significantly less blood loss, fewer incisional infections or febrile episodes, shorter hospital stays and speedier return to normal activities than those who underwent laparotomy [8]. Power morcellation plays an important role in the extraction of large leiomyoma from the abdominal cavity during minimally invasive surgery. However, following reports of poor outcomes in patients with inadvertently morcellated uterine sarcoma, the FDA has discouraged the use of laparoscopic power morcellation during hysterectomy or myomectomy for uterine fibroids.

Based on the literature, the FDA has reported that one in 352 women have unsuspected uterine sarcoma while undergoing surgery for presumed benign disease [3]. Sarcoma prevalence estimates are highly dependent on age, with the lowest prevalence among women under the age of 50 and the highest prevalence among women older than 60 [9]. As myomectomies are usually performed in the younger age group, the actual incidence of uterine sarcoma after myomectomy would appear to be lower than expected. The risk of cancer in women who undergo myomectomy performed using power morcellation is lower than that reported for hysterectomy. The prevalence of uterine cancer has been found to be 0.19 % (1 in 528) in women who undergo myomectomy without morcellation and 0.09 % (1 in 1073) in those who undergo power morcellation [10]. Therefore, in patients with unexpected uterine sarcoma after uterus-preserving surgery, tumor aggression resulting from initial surgery without power morcellation may be more common than tumor dissemination with power morcellation.

Recent National Comprehensive Cancer Network (NCCN) guidelines recommend en bloc tumor resection without tumor disruption as the standard treatment for localized sarcoma, which is consistent with the accepted management principles for soft tissue sarcoma arising in any anatomical location [11]. In uterus-preserving surgery, there may be concerns about tumor disruption associated with surgery or any remaining tumor. Therefore, patients who have undergone uterus-preserving surgery for presumed benign uterine disease and are found to have sarcoma on final pathology represent a management dilemma. However, few studies assess the prognosis for unexpected uterine sarcoma after myomectomy [4, 5].

Zhang et al. reported the outcomes of nine patients with unexpected uterine sarcoma after myomectomy [5]. Eight patients underwent a secondary operation, and endometrial stromal sarcoma was the dominant subtype of unexpected uterine sarcoma in the study. All patients were alive and there was only one case of local recurrence in the preserved ovary. Cusido et al. reported no significant difference in prognosis for uterine sarcoma in terms of myomectomy versus hysterectomy as the initial surgery [4]. Of the 14 patients who underwent myomectomy in this study, eight (57 %) underwent a secondary operation with hysterectomy. In terms of PFS, no statistical differences were found in our study. In addition, our results suggest that there may be benefits to surgical re-exploration. Of the patients who underwent re-exploration at a referral institution after myomectomy or subtotal hysterectomy, approximately 35.8 % had remnant sarcoma on the remaining uterus. However, the value of lymphadenectomy, appendectomy or omentectomy for identifying occult metastasis in early-stage uterine sarcoma appears to be low.

Regarding morcellation, our results are consistent with previous studies showing poorer outcomes with morcellation in uterine sarcoma. Concerns have been raised as uterine morcellation carries a risk of disseminating unexpected malignancy with an apparent associated increase in mortality [12]. Previous studies have shown that morcellation has a negative impact on survival outcomes in uterine sarcoma [12, 13]. Taking into consideration the negative impact of morcellation in sarcomas, this technique should be used with caution in patients with suspicious uterine sarcoma.

## Conclusions

Initial uterus-preserving surgery does not appear to be associated with an adverse impact on survival outcomes for unexpected uterine sarcoma when surgical re-exploration is performed immediately. In our opinion, myomectomy or subtotal hysterectomy remain the preferred options for treating women with presumed leiomyoma who want to preserve the uterus as no difference in survival was found between uterus-preserving surgery and total hysterectomy, even in cases with unexpected sarcoma. Our second recommendation is that patients who undergo myomectomy or subtotal hysterectomy where uterine sarcoma is detected on final pathology undergo immediate surgical re-exploration. Surgical re-exploration appears to be useful for removing remnant sarcoma in the remaining myometrium, which is a key factor for improving outcomes in this disease subset. Further large-scale studies are required to document the outcomes of unexpected uterine sarcoma with or without morcellation after uterus-preserving surgery.

## Additional file

Additional file 1: Figure S1. Progression-free survival of patients with unexpected sarcoma stratified by morcellation procedure. **Figure S2.** Progression-free survival of patients with unexpected sarcoma stratified by morcellation procedure in myomectomy/subtotal hysterectomy group. **Figure S3.** Progression-free survival of patients with unexpected sarcoma stratified by type of initial surgery in myomectomy/subtotal hysterectomy group. (PPTX 70 kb)
