# Supplementary material for: Outcomes of uterine sarcoma found incidentally after uterus-preserving surgery for presumed benign disease
Source: BMC Cancer. 2016 Aug 23;16(1):675. doi: 10.1186/s12885-016-2727-x (PMC4995644; doi:10.1186/s12885-016-2727-x)
Supplement: Additional file 1: Figure S1. — Progression-free survival of patients with unexpected sarcoma stratified by morcellation procedure. Figure S2. Progression-free survival of patients with unexpected sarcoma stratified by morcellation procedure in myomectomy/subtotal hysterectomy group. Figure S3. Progression-free survival of patients with unexpected sarcoma stratified by type of initial surgery in myomectomy/subtotal hysterectomy group. (PPTX 70 kb) [file 12885_2016_2727_MOESM1_ESM.pptx]

## Slide 1
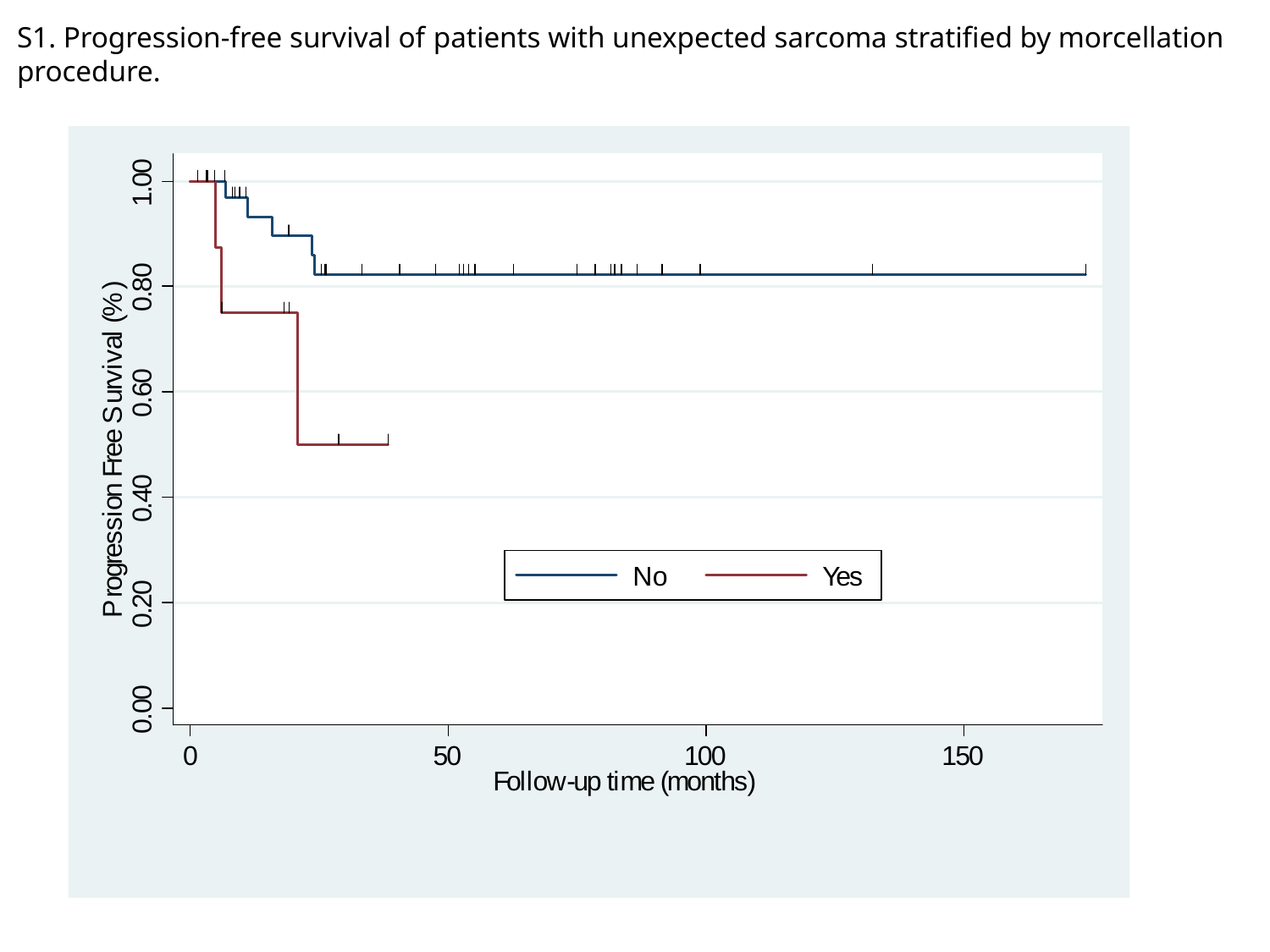

S1. Progression-free survival of patients with unexpected sarcoma stratified by morcellation procedure.

## Slide 2
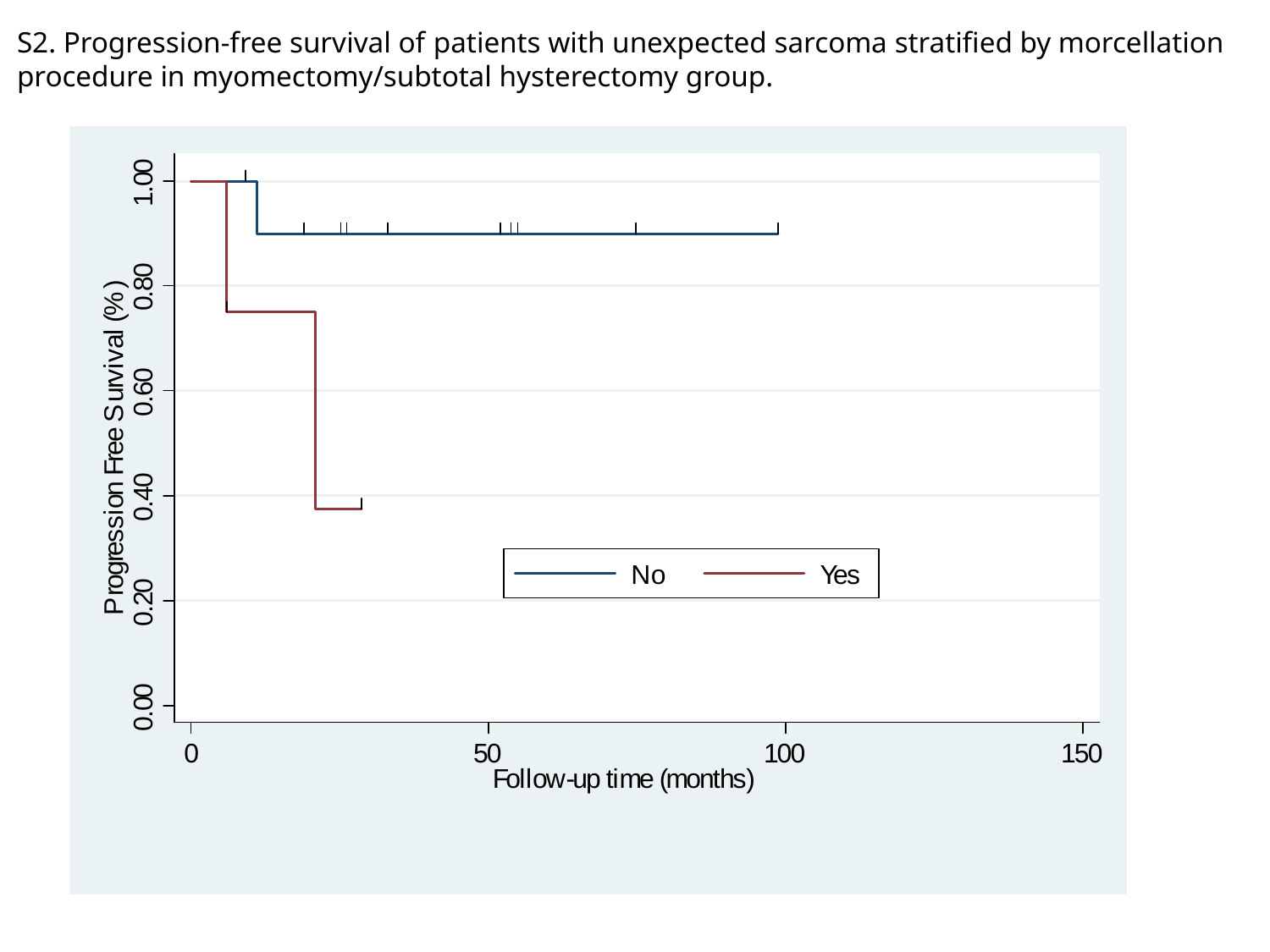

S2. Progression-free survival of patients with unexpected sarcoma stratified by morcellation procedure in myomectomy/subtotal hysterectomy group.

## Slide 3
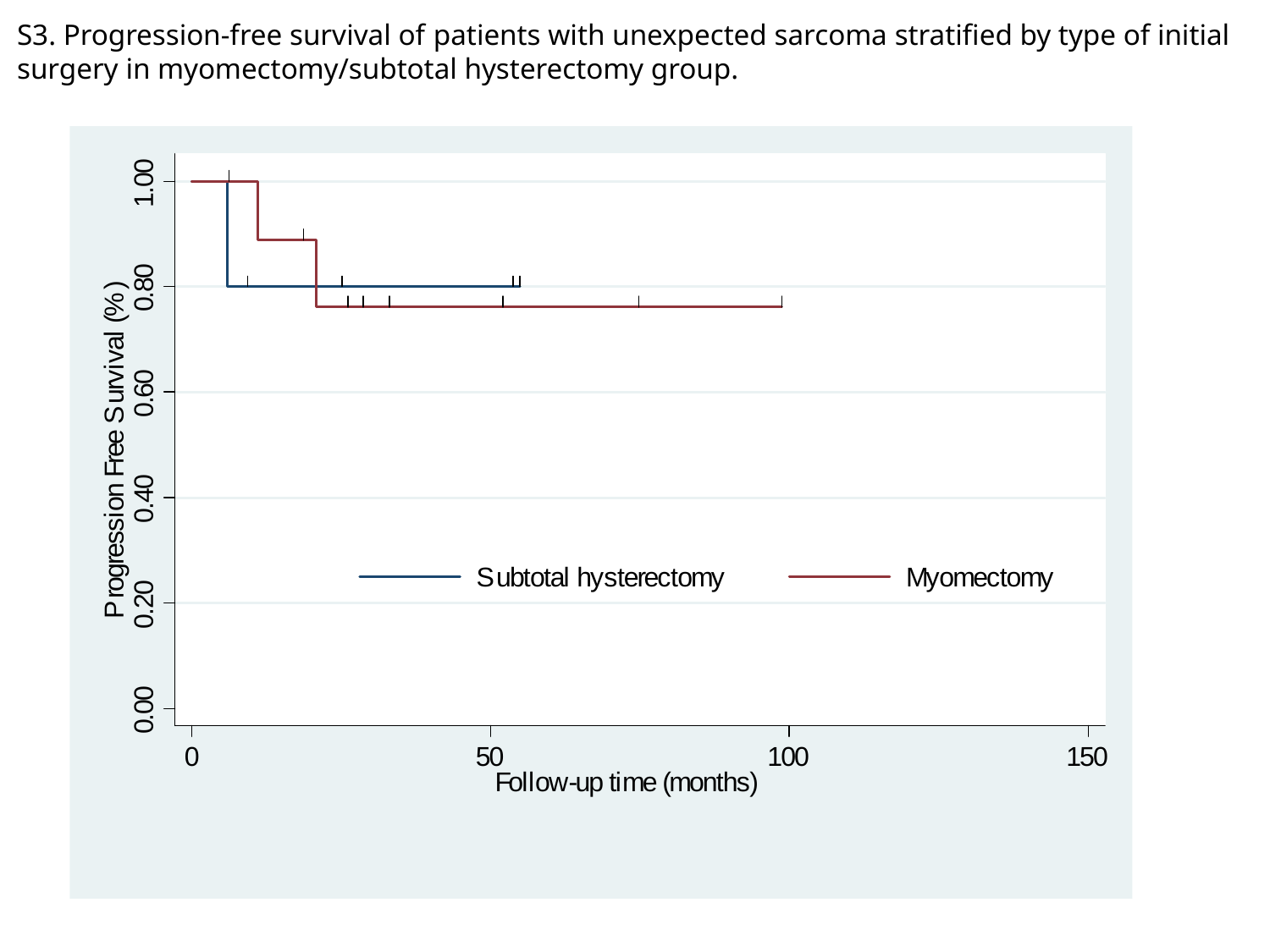

S3. Progression-free survival of patients with unexpected sarcoma stratified by type of initial surgery in myomectomy/subtotal hysterectomy group.
